# A novel role of sesamol in inhibiting NF-κB-mediated signaling in platelet activation

**DOI:** 10.1186/1423-0127-18-93

**Published:** 2011-12-14

**Authors:** Chao-Chien Chang, Wan-Jung Lu, Eng-Thiam Ong, Cheng-Wen Chiang, Song-Chow Lin, Shih-Yi Huang, Joen-Rong Sheu

**Affiliations:** 1 Graduate Institute of Clinical Medicine, Taipei Medical University, 250 Wu-Hsing St., Taipei 11031, Taiwan; 2 Department of Cardiology, Cathay General Hospital, 280 Renai Rd. Sec.4, Taipei 10630, Taiwan; 3 Department of Pharmacology, School of Medicine, Taipei Medical University, 250 Wu-Hsing St., Taipei 11031, Taiwan; 4 School of Nutrition and Health Sciences, Taipei Medical University, 250 Wu-Hsing St., Taipei 11031, Taiwan

**Keywords:** IκBα, IKK, intracellular Ca^2+^, protein kinase A, platelet activation, sesamol

## Abstract

**Background:**

Platelet activation is relevant to a variety of coronary heart diseases. Our previous studies revealed that sesamol possesses potent antiplatelet activity through increasing cyclic AMP formation. Although platelets are anucleated cells, they also express the transcription factor, NF-κB, that may exert non-genomic functions in platelet activation. Therefore, we further investigated the inhibitory roles of sesamol in NF-κB-mediated platelet function.

**Methods:**

Platelet aggregation, Fura 2-AM fluorescence, and immunoblotting analysis were used in this study.

**Results:**

NF-κB signaling events, including IKKβ phosphorylation, IκBα degradation, and p65 phosphorylation, were markedly activated by collagen (1 μg/ml) in washed human platelets, and these signaling events were attenuated by sesamol (2.5~25 μM). Furthermore, SQ22536 and ODQ, inhibitors of adenylate cyclase and guanylate cyclase, respectively, strongly reversed the sesamol (25 μM)-mediated inhibitory effects of IKKβ phosphorylation, IκBα degradation, and p65 phosphorylation stimulated by collagen. The protein kinase A (PKA) inhibitor, H89, also reversed sesamol-mediated inhibition of IκBα degradation. Moreover, BAY11-7082, an NF-κB inhibitor, abolished IκBα degradation, phospholipase C (PLC)γ2 phosphorylation, protein kinase C (PKC) activation, [Ca^2+^]i mobilization, and platelet aggregation stimulated by collagen. Preincubation of platelets with the inhibitors, SQ22536 and H89, both strongly reversed sesamol-mediated inhibition of platelet aggregation and [Ca^2+^]i mobilization.

**Conclusions:**

Sesamol activates cAMP-PKA signaling, followed by inhibition of the NF-κB-PLC-PKC cascade, thereby leading to inhibition of [Ca^2+^]i mobilization and platelet aggregation. Because platelet activation is not only linked to hemostasis, but also has a relevant role in inflammation and metastasis, our data demonstrating that inhibition of NF-κB interferes with platelet function may have a great impact when these types of drugs are considered for the treatment of cancer and various inflammatory diseases.

## Background

Sesamol (3,4-methylenedioxyphenol) is a constituent of roasted sesame seeds, *Sesamum indicum *L., an important oilseed crop [1]. Sesamol is a potent phenolic antioxidant contained only in processed sesame oil. Several beneficial effects of sesamol were reported including antioxidation, chemoprevention, antimutagenic, and antihepatotoxic properties [2-5]. Traditionally, sesame seed oil was used to remove wrinkles and prevent aging, when applied in a facial massage to the skin [5]. Recently, sesamol was found to induce growth arrest and apoptosis in cancer and cardiovascular cells [6]. Sesamol was also found to enhance vascular fibrinolytic capacity through regulating gene expression of a plasminogen activator and nitric oxide (NO) release in endothelial cells [7,8].

Arterial thrombosis is quite distinct from venous thrombosis in that arterial thrombosis is mostly composed of platelets that adhere to ruptured endothelial surfaces [9]. Venous thrombosis, which is enriched in fibrin and erythrocytes, can occur in the absence of vessel wall damage. Therefore, platelet aggregation may play a crucial role in the atherothrombotic process [10].

Despite the very important roles of platelets in the development of acute thrombosis, coronary heart diseases (CHDs), and atherosclerosis, no data are available concerning the effect of sesamol on platelet activation. Recently, we reported that sesamol exhibited potent activity of inhibiting platelet aggregation [11]. Its mechanism may involve an increase in the cAMP-endothelial NO synthase (eNOS)/NO-cGMP pathway, followed by inhibition of the phospholipase Cγ2 (PLCγ2)-protein kinase C (PKC)-p38 mitogen-activated protein kinase (MAPK)-thromboxane A_2 _cascade, thereby leading to inhibition of [Ca^2+^]i mobilization, and finally inhibition of platelet aggregation [11].

In the present study, we further investigated the mechanisms of sesamol in inhibiting platelet activation in greater detail, and found that sesamol obviously suppressed nuclear factor-kappa B (NF-κB)-mediated signaling events in washed human platelets. NF-κB, a transcription factor, regulates diverse cell functions ranging from inflammation to cell death. As the term, "nuclear factor" implies, the actions of NF-κB require its translocation from the cytosol to the nucleus to bind cognate nuclear DNA sequences. Platelets are anucleated, do not differentiate or proliferate, and thus are a good model for studying non-genomic functions of NF-κB in sesamol-mediated inhibition of NF-κB activation. We therefore for the first time examined the cellular NF-κB signaling events associated with sesamol-mediated inhibition of platelet activation.

## Methods

### Materials

Sesamol, collagen (type I), prostaglandin E_1 _(PGE_1_), heparin, (E)-3-(4-methylphenylsulfonyl)-2-propenenitrile (BAY11-7082), 9-(tetrahydro-2-furanyl)-9H-purin-6-amine (SQ22536), N-[2-(p-bromocinnamylamino)ethyl]-5-isoquinolinesulfonamide dihydrochloride (H89), and 1H-[1,2,4]oxadiazolo[4,3-a]quinoxalin-1-one (ODQ) were purchased from Sigma Chemical (St Louis, MO, USA); Fura 2-AM was from Molecular Probe (Eugene, OR, USA); the anti-phospho-IKKα (Ser^180^)/IKKβ (Ser^181^) polyclonal antibody (pAb), anti-IκBα (44D4) pAb, anti-PLCγ2, anti-phospho (Tyr^759^) PLCγ2 monoclonal antibodies (mAbs), anti-phospho (Ser) PKC substrate (p47) pAb, and the anti-phospho-NF-κB p65 (Ser^536^) pAb were from Cell Signaling (Beverly, MA, USA); the anti-α-tubulin mAb was from NeoMarkers (Fremont, CA, USA); and the Hybond-P polyvinylidene difluoride (PVDF) membrane, enhanced chemiluminescence (ECL) Western blotting detection reagent and analysis system, horseradish peroxidase (HRP)-conjugated donkey anti-rabbit immunoglobulin G (IgG), and sheep anti-mouse IgG were from Amersham (Buckinghamshire, UK). Sesamol was dissolved in 0.5% dimethyl sulfoxide (DMSO) and stored at 4°C until used.

### Platelet aggregation

Human platelet suspensions were prepared as previously described [10]. This study was conducted according to the guidelines laid down in the Declaration of Helsinki and all procedures involving human subjects were approved by the Institutional Review Board of Taipei Medical University, and all human volunteers provided informed consent. In brief, blood was collected from healthy human volunteers who had taken no medicine during the preceding 2 weeks, and was mixed with acid/citrate/glucose (9:1; v/v). After centrifugation at 120 *g *for 10 min, the supernatant (platelet-rich plasma; PRP) was supplemented with PGE_1 _(0.5 μM) and heparin (6.4 IU/ml), and then incubated for 10 min at 37°C and centrifuged at 500 *g *for 10 min. The platelet pellets were suspended in 5 ml of Tyrode's solution, pH 7.3 [containing (mM) NaCl 11.9, KCl 2.7, MgCl_2 _2.1, NaH_2_PO_4 _0.4, NaHCO_3 _11.9, and glucose 11.1], then apyrase (1.0 U/ml), PGE_1 _(0.5 μM), and heparin (6.4 IU/ml) were added, and the mixture was incubated for 10 min at 37°C. After centrifugation of the suspensions at 500 *g *for 10 min, the washing procedure was repeated. The washed platelets were finally suspended in Tyrode's solution containing bovine serum albumin (BSA) (3.5 mg/ml) and adjusted to about 4.5 × 10^8 ^platelets/ml. The final concentration of Ca^2+ ^in the Tyrode's solution was 1 mM.

A turbidimetric method was applied to measure platelet aggregation [10], using a Lumi-Aggregometer (Payton, Scarborough, Ontario, Canada). Platelet suspensions (3.6 × 10^8 ^platelets/ml) were preincubated with various concentrations of sesamol or inhibitors for 3 min before the addition of collagen (1 μg/ml). The reaction was allowed to proceed for 6 min, and the extent of aggregation was expressed in light-transmission units.

### Measurement of platelet [Ca^2+^]i mobilization by Fura 2-AM fluorescence

Citrated whole blood was centrifuged at 120 *g *for 10 min. The PRP was incubated with Fura 2-AM (5 μM) for 1 h. Washed platelets (8 × 10^8 ^platelets/ml) were then prepared as described above. Finally, the external Ca^2+ ^concentration of the platelet suspensions was adjusted to 1 mM. The rise in the [Ca^2+^]i was measured using a fluorescence spectrophotometer (CAF 110, Jasco, Tokyo, Japan) with excitation wavelengths of 340 and 380 nm, and an emission wavelength of 500 nm [10].

### Immunoblotting study

Washed platelets (1.2 × 10^9^/ml) were preincubated with sesamol (2.5~25 μM) or various inhibitors for 3 min, followed by the addition of collagen (1 μg/ml) to trigger platelet activation. The reaction was stopped, and platelets were immediately re-suspended in 200 μl of lysis buffer (50 mM Hepes, 5 mM EDTA, 50 mM NaCl, 1% triton X-100, 10 μg/ml aprotinin, 1 mM phenylmethylsulfonylfluoride, 10 μg/ml leupeptin, 10 mM NaF, 1 mM sodium orthovanadate, 5 mM sodium pyrophosphate, and 2 mM dithiothreitol) for 1 h. Lysates were centrifuged at 5000 *g *for 5 min. Samples containing 80 μg of protein were separated by sodium dodecylsulfate polyacrylamide gel electrophoresis (SDS-PAGE) (12%); proteins were electrotransferred by a semidry transfer method (Bio-Rad, Hercules, CA). Blots were blocked with TBST (10 mM Tris-base, 100 mM NaCl, and 0.01% Tween 20) containing 5% BSA for 1 h and then probed with various primary antibodies (diluted 1:1000 in TBST). Membranes were incubated with HRP-linked anti-mouse IgG or anti-rabbit IgG (diluted 1:3000 in TBST) for 1 h. Immunoreactive bands were detected by an ECL system. The bar graph depicts the ratios of semiquantitative results obtained by scanning reactive bands and quantifying the optical density using videodensitometry (Bio-profil; Biolight Windows Application V2000.01; Vilber Lourmat, France).

### Determination of lactate dehydrogenase (LDH)

In brief, washed platelets (3.6 × 10^8^/ml) were preincubated with Tyrode's solution, solvent control (0.5% DMSO), and various concentrations of sesamol (5~100 μM) for 20 min at 37°C, a 10-μl aliquot of supernatant was deposited on a Fuji Dri-Chem slide LDH-PIII (Fuji, Tokyo, Japan), and the absorbance wavelength was read at 540 nm using an ultraviolet-visible recording spectrophotometer (UV-160; Shimazu, Japan). A maximal value (MAX) of LDH was observed from sonicated platelets.

### Data analysis

The experimental results are expressed as the means ± S.E.M. and are accompanied by the number of observations (n). Values of *n *refer to the number of experiments, each made with different blood donors. All experiments were assessed by an analysis of variance (ANOVA). If this analysis indicated significant differences among group means, then each group was compared using the Newman-Keuls method. *p <*0.05 was considered statistically significant.

## Results

### Concentration- and time-dependent effects of sesamol on collagen-induced NF-κB activation in washed human platelets

**Figure 1:**
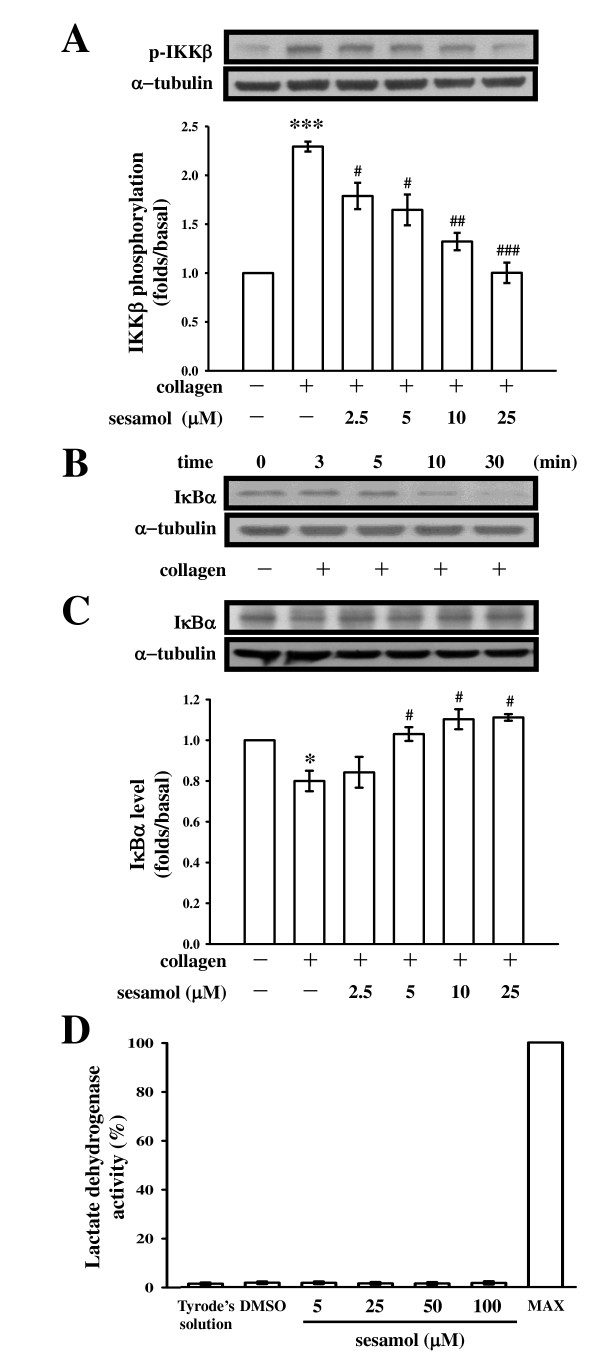
**Effects of sesamol on IKKβ phosphorylation and IκBα degradation in collagen-activated platelets**. Washed platelets (1.2 × 10^9^/ml) were preincubated with or without sesamol (2.5~25 μM) or a solvent control (0.5% DMSO), followed by the addition of collagen (1 μg/ml) to trigger (**A**) IKKβ phosphorylation for 3 min or (**B**) IκBα degradation for the indicated times (3~30 min), and (**C**) IκBα degradation for 10 min. Cells were collected, and subcellular extracts were analyzed as described in "Methods". For the lactate dehydrogenase (LDH) experiment (**D**), washed platelets (3.6 × 10^8^/ml) were preincubated with Tyrode's solution, solvent control (0.5% DMSO), and various concentrations of sesamol (5~100 μM) for 20 min at 37°C, a 10-μl aliquot of supernatant was deposited on a Fuji Dri-Chem slide LDH-PIII as described in "Methods". Data are presented as the means ± S.E.M. (n = 4). *p <0.05 and ***p <0.001, compared to the control group (resting); ^#^*p <*0.05, ^##^*p <*0.01, and ^###^*p <*0.001, compared to the collagen group.

In our previous report [11], sesamol (1~5 μM) exhibited potent activity of inhibiting platelet aggregation stimulated by collagen (1 μg/ml); it also significantly inhibited platelet aggregation stimulated by arachidonic acid (AA) (60 μM) at higher concentrations (5~10 μM). In the present study, we used collagen as an agonist to further explore the inhibitory mechanisms of sesamol in platelet activation. The pleiotropic NF-κB normally exists as an inactive cytoplasmic complex, the predominant form of which is a heterodimer composed of p50 and p65 subunits tightly bound to inhibitory proteins of the IκB family [12]. Once phosphorylated by the IκB kinase (IKK) complex, IκB dissociates from NF-κB subunits and is ubiquitinated and rapidly degraded by the proteasome [12]. IKK phosphorylation was proposed as being a major mode for IκBα degradation, leading to NF-κB activation [12]. As shown in Figure 1A, IKKβ phosphorylation significantly increased 3 min after being stimulated by collagen (1 μg/ml) in washed platelets. The compiled data of Figure 1A are shown at the bottom. Pretreatment of platelets with various concentrations of sesamol (2.5~25 μM) concentration-dependently attenuated collagen-induced IKKβ phosphorylation (Figure 1A). Based on the above results indicating that sesamol's attenuation of IKKβ phosphorylation may interfere with the IKK-IκBα cascade, we sought to further examine whether sesamol interferes with IκBα degradation. Treatment with collagen caused the rapid, time-dependent disappearance of the immunoreactive bands of IκBα (Figure 1B). The IκBα protein was markedly degraded within 10 min, and reached maximal degradation at 30 min after collagen stimulation. Sesamol (2.5~25 μM) concentration-dependently reversed the degradation of IκBα protein at 10 min after collagen stimulation (Figure 1C). These results suggest that IKKβ phosphorylation and subsequent IκBα degradation in collagen-stimulated platelets may contribute to sesamol's inhibitory actions on NF-κB signaling. In addition, the lactate dehydrogenase (LDH) study revealed that sesamol (5~100 μM) incubated with platelets for 20 min did not significantly increase LDH activity, even at a higher concentration (100 μM) (Figure 1D), indicating that sesamol did not affect platelet permeability or induce platelet cytolysis, it clearly shows that no cytotoxic effects of sesamol on platelets at these concentrations.

### The roles of cyclic nucleotides in sesamol-mediated inhibition of NF-κB signaling

**Figure 2:**
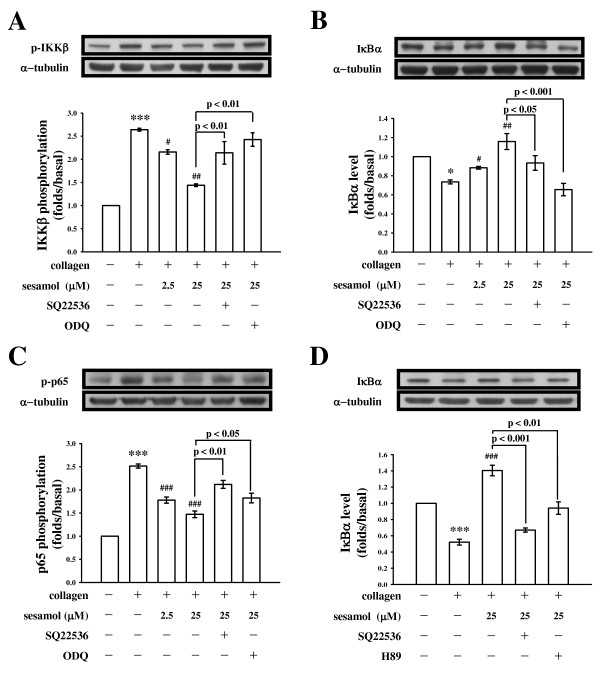
**Cyclic nucleotide-dependent inhibition of IKKβ phosphorylation, IκBα degradation, and p65 phosphorylation by sesamol in collagen-activated platelets**. Washed platelets (1.2 × 10^9^/ml) were preincubated with sesamol (2.5 and 25 μM) or a solvent control (0.5% DMSO) in the absence or presence of SQ22536 (100 μM), ODQ (20 μM), or H89 (5 μM), followed by the addition of collagen (1 μg/ml) to trigger (**A**) IKKβ phosphorylation for 3 min or (**B **and **D**) IκBα degradation and (**C**) p65 phosphorylation for 10 min. Cells were collected, and subcellular extracts were analyzed by immunoblotting. Data are presented as the means ± S.E.M. (n = 4). *p <0.05 and ***p <0.001, compared to the control group (resting); ^#^*p <*0.05, ^##^*p <*0.01, and ^###^*p <*0.001, compared to the collagen group.

In our previous description [11], sesamol increased levels of both cAMP and cGMP, suggesting that increased cAMP stimulated eNOS activity and NO biosynthesis, followed by increasing cGMP formation. cAMP is the upstream regulator of the eNOS-NO-cGMP cascade in sesamol-mediated antiplatelet effects. To investigate whether sesamol-mediated inhibition of NF-κB activation was also regulated by cyclic nucleotides, especially cAMP, we used two different cyclic nucleotide inhibitors, SQ22536 (100 μM) that inhibits adenylate cyclase and ODQ (20 μM) an inhibitor of guanylate cyclase. Both inhibitors strongly reversed the sesamol (25 μM)-mediated inhibition of IKKβ phosphorylation (Figure 2A) and IκBα degradation (Figure 2B) stimulated by collagen in washed platelets. In addition, Liu *et al. *[13] showed that platelets express three members of the NF-κB pathway: IKK, IκB, and NF-κB p65. The present study also demonstrated that p65 phosphorylation was markedly increased, and sesamol (2.5 and 25 μM) concentration-dependently attenuated p65 phosphorylation in platelets stimulated by collagen (1 μg/ml) (Figure 2C). In the presence of SQ22536 (100 μM) and ODQ (20 μM), both inhibitors clearly reversed the sesamol (25 μM)-mediated inhibition of p65 phosphorylation (Figure 2C).

The effects of cyclic nucleotides are mediated via their respective protein kinase (i.e., PKA, a specific cAMP-dependent protein kinase), which phosphorylates substrate proteins involved in platelet inhibitory pathways [14]. To investigate whether sesamol's inhibition of NF-κB was regulated by PKA, a PKA inhibitor (H89) that inhibits ATP binding to PKA catalytic subunits (PKAc) was used. As shown in the Figure 2D, H89 (5 μM) exhibited a similar effect as SQ22536 (100 μM) in reversing the sesamol-mediated inhibition of IκBα degradation.

### The roles of NF-κB in regulating the PLCγ2-PKC cascade in platelets

**Figure 3:**
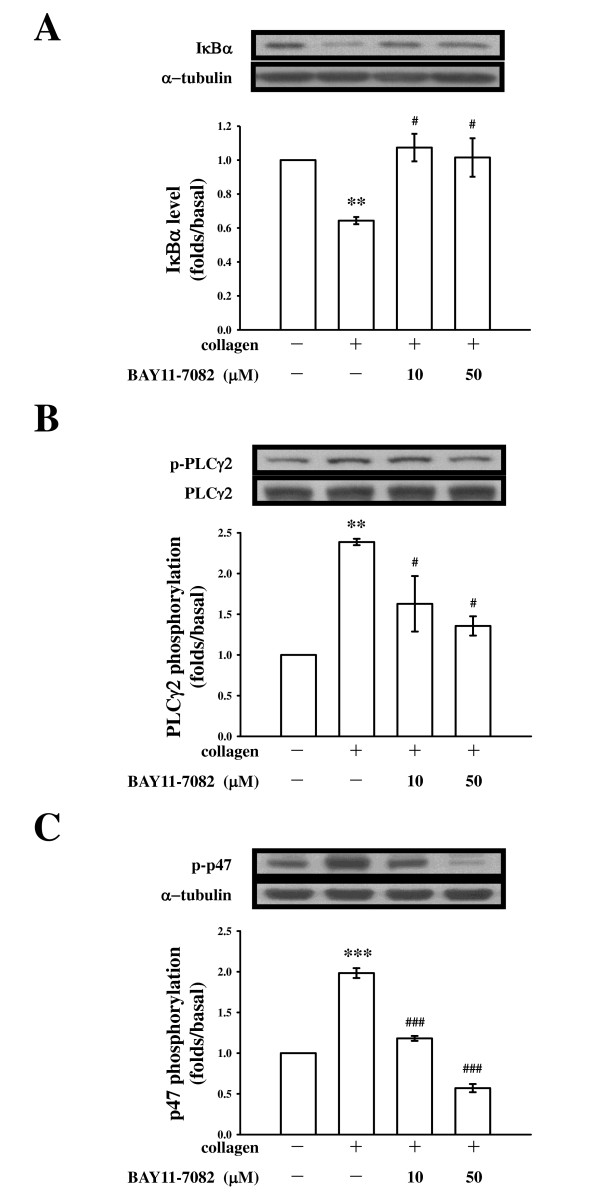
**NF-κB regulates phospholipase Cγ2 (PLCγ2) phosphorylation, and protein kinase C (PKC) activity in collagen-activated platelets**. Washed platelets (1.2 × 10^9^/ml) were preincubated with or without BAY11-7082 (10 and 50 μM), followed by the addition of collagen (1 μg/ml) to trigger (**A**) IκBα degradation for 10 min or (**B**) PLCγ2 and (**C**) PKC substrate (p47) phosphorylation for 5 min. Cells were collected, and subcellular extracts were analyzed by immunoblotting. Data are presented as the means ± S.E.M. (n = 4). **p <0.01 and ***p <0.001, compared to the control group (resting); ^#^*p <*0.05 and ^###^*p <*0.001, compared to the collagen group.

As described previously [11], we suggest that sesamol may increase the level of cAMP, followed by inhibition of the PLCγ2-PKC cascade, thereby leading to inhibition of [Ca^2+^]i mobilization, and finally inhibition of platelet aggregation. PLC hydrolyzes phosphatidylinositol 4,5-bisphosphate (PIP_2_) to generate two secondary messengers: inositol 1,4,5-trisphosphate (IP_3_) and diacylglycerol (DAG) [15]. DAG activates PKC, inducing 40~47-kDa protein phosphorylation. To further establish the cellular signaling events of NF-κB associated with the PLCγ2-PKC cascade in sesamol-mediated inhibition of platelet activation, an NF-κB inhibitor, BAY11-7082, which is an irreversible inhibitor of IκBα phosphorylation was used [16]. The immunoblotting analysis revealed that treatment with BAY11-7082 (10 and 50 μM) concentration-dependently abolished IκBα degradation (Figure 3A) and PLCγ2 phosphorylation (Figure 3B) stimulated by collagen (1 μg/ml). When collagen was added to human platelets, a protein with an apparent molecular weight of 47 kDa (p47) was predominately phosphorylated compared to resting platelets (Figure 3C). BAY11-7082 (10 and 50 μM) also abolished p47 phosphorylation, indicating that NF-κB can regulate PLC-PKC signaling in platelets.

### The functional relevance of NF-κB in [Ca^+2^]i mobilization and platelet aggregation

**Figure 4:**
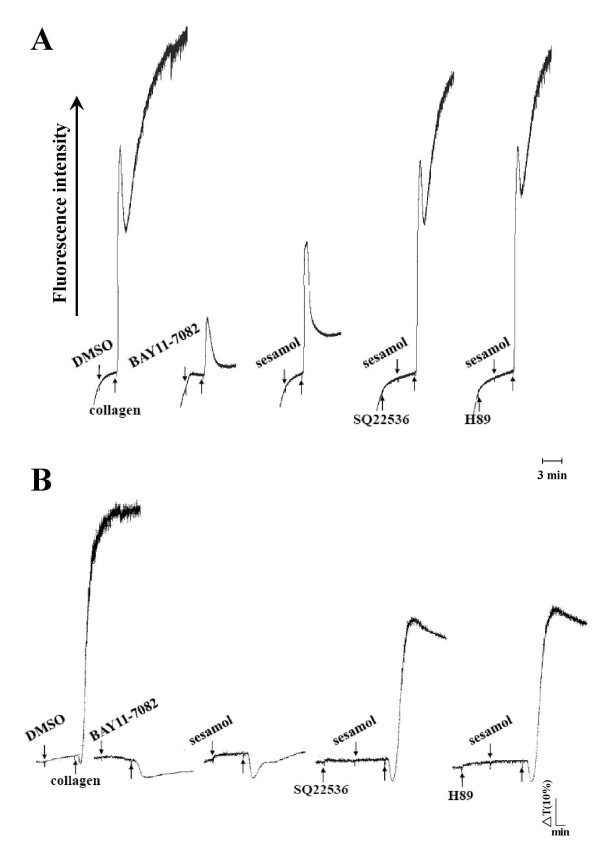
**Effects of sesamol on the inhibition of [Ca**^**2+**^**]i mobilization and platelet aggregation in the presence of various inhibitors in collagen-activated platelets**. Washed platelets were preincubated with sesamol (25 μM) or 0.5% DMSO in the presence of BAY11-7082 (10 μM), SQ22536 (100 μM), or H89 (5 μM), followed by the addition of collagen (1 μg/ml) to trigger (**A**) [Ca^2+^]i mobilization and (**B**) platelet aggregation for 6 min as described in "Methods". The profiles (**A **and **B**) are representative examples of four similar experiments.

To demonstrate the physiological relevance of NF-κB in platelet activation, we investigated effects of NF-κB inhibitors on [Ca^2+^]i mobilization and platelet aggregation. BAY11-7082, at low concentration of up to 10 μM, not only significantly attenuated [Ca^2+^]i mobilization, but also inhibited platelet aggregation stimulated by collagen (Figure 4A, B). In addition, preincubation of platelets with the inhibitors, SQ22536 (100 μM) and H89 (5 μM), both strongly reversed sesamol's inhibition of [Ca^2+^]i mobilization and platelet aggregation (Figure 4A, B). Taken together, our data suggest that NF-κB is involved in [Ca^2+^]i mobilization and platelet aggregation, and sesamol's inhibition of NF-κB is mediated, at least partly, by a cyclic nucleotide-dependent pathway.

## Discussion

The function of NF-κB has been extensively studied in nucleated cells. Diverse stimuli, including cytokines, viral infection, UV radiation, and free radicals, can induce NF-κB activation. Genes regulated by NF-κB include those involved in inflammation, cell survival, differentiation, and proliferation responses [12]. Therefore, NF-κB is an attractive target for therapeutic interventions against cancer and inflammatory diseases. Platelets are anucleated cells; however, several studies found that platelets express transcription factors such as steroid/nuclear receptors [17], a glucocorticoid receptor [18], and peroxisome proliferator-activated receptors (PPARs) [19]. Those findings suggest that transcription factors can exert non-genomic functions on platelets.

**Figure 5:**
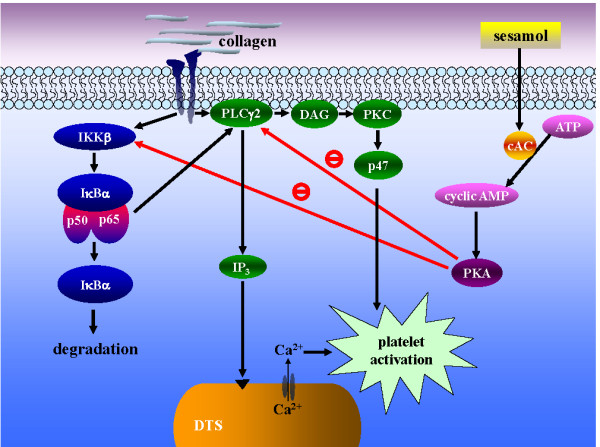
**The hypothesis of inhibitory signaling of sesamol in platelet activation**. Collagen binds to its receptors, and then activates both the PLCγ2-DAG-PKC and NF-κB pathways. Activated phospholipase Cγ2 (PLCγ2) catalyses phosphatidylinositol 4,5-bisphosphate (PI4,5-P_2_) into 1,2-diacylglycerol (DAG) and inositol 1,4,5-trisphosphate (IP_3_). DAG activates protein kinase C (PKC), followed by phosphorylation of a 47-kDa protein (p47). IP_3 _induces the release of Ca^2+ ^from the dense tubular system (DTS). On the other hand, IKKβ activates NF-κB signaling including IκBα degradation and p65 phosphorylation, which further activates the PLCγ2-PKC pathway. Sesamol can activate cyclic AMP-protein kinase A (PKA), followed by inhibition of both the PLCγ2 and NF-κB cascade (i.e., IKKβ), and finally inhibits platelet activation.

It was shown that IκBα is phosphorylated and degraded after platelet activation [13]. The functional significance of NF-κB is still not clear. The question remains as to whether or not this transcription factor is functionally present in a novel way, unrelated to transcriptional regulation, in anucleated platelets. It was demonstrated that pretreatment with an NF-κB inhibitor prevented multiple platelet activation mechanisms, such as platelet adhesion to fibrinogen, integrin α_IIb_β_3 _activation, P-selectin expression, and thromboxane A_2 _(TxA_2_) formation [20]. NF-κB signaling also inhibited the phosphorylation of extracellular signal-regulated kinases (ERKs), which regulate cytosolic phospholipase A_2 _(cPLA_2_) activity, the main enzyme responsible for the release of AA, which is converted to form TxA_2 _in platelets [20]. Moreover, three IKK (α, β, and γ) family members are expressed in platelets, with the β form being even more strongly expressed in platelets than either α or γ form. In the present study, IKKβ phosphorylation, IκBα degradation, and p65 phosphorylation were also observed in collagen-activated platelets; our results are consistent with those of previous studies [13,20]. Furthermore, pretreatment with the NF-κB inhibitor, BAY11-7082, clearly attenuated PLC-PKC activation, [Ca^2+^]i mobilization, and platelet aggregation (Figures 3 and 4). PLC is a key enzyme in signal transduction [21]. There are six major families of PLC enzymes which consist of at least 13 PLC isoforms: PLCβ (1~4), PLCγ (1 and 2), PLCδ (1, 3, and 4), PLCε (1), PLCζ (1), and PLCη (1 and 2) [21]. PLCγ2 is involved in antigen-dependent signaling in B cells and collagen-dependent signaling in platelets [22]. Activation of PLCγ2 results in the degradation of phosphoinositides, notably, phosphatidylinositol 4,5-bisphosphate (PI4,5-P_2_), resulting in the production of the second messengers, IP_3 _and DAG [23]. IP_3 _triggers an increase in intracellular Ca^2+ ^from Ca^2+^-storage sites (i.e., the dense tubular system, DTS) in platelets. DAG activates PKC-inducing protein phosphorylation (p47) (Figure 5). PKC activation represents a strategy adopted by cells to allow selected responses to specific activating signals in distinct cellular compartments [24]. Our results suggest that NF-κB may be a novel upstream regulator of the PLC-PKC cascade in activated platelets (Figure 5). These data indicate that NF-κB transcription factors could have functions other than regulating gene expression and that anucleated platelets are a relevant model for investigating these functions. Furthermore, our data do not exclude other potential, yet-unidentified functions of NF-κB family members in platelets.

In human platelets, cAMP or cGMP plays a crucial role in platelet inhibition. The effect of cAMP is mediated via cAMP-dependent protein kinase (PKA). PKA is a tetrameric holoenzyme consisting of a regulatory (PKAr) subunit dimer and two catalytic (PKAc) subunits. Elevation of cAMP levels and binding of cAMP to PKAr causes dissociation of the kinase complex and release of free active catalytic subunits [25]. Although PKA is mainly activated by cAMP, a fraction of total cellular PKA forms a complex with NF-κB-IκB proteins and may be released upon NF-κB activation by different stimuli [26,27]. Recently, Gambaryan *et al. *[27] have reported that PKA is also activated through cAMP-independent mechanisms, which involves to be dissociated the PKAc from NF-κB-IκB-PKAc complex by triggering IKKβ phosphorylation in thrombin- and collagen-activated platelets. This effect is taken as a novel feedback inhibitory mechanism for prevention of undesired platelet activation. In a previous study [11], we showed that sesamol increases cAMP formation and phosphorylates vasodilator-stimulated phosphoprotein (VASP), which was obviously reversed in the presence of SQ22536. In the present study, sesamol markedly inhibited NF-κB activation (i.e., IKKβ phosphorylation) (Figure 1) in collagen-stimulated platelets. These results suggested that sesamol activates PKA through a classical cAMP-dependent mechanism, which phosphorylates substrate proteins involved in platelet inhibitory pathways. Herein, we propose a novel platelet inhibitory pathway of inhibiting NF-κB activation by cAMP/PKA (Figure 5). However, our experiments do not completely rule out the possibility that other, yet-unidentified kinases besides cAMP/PKA are involved in sesamol-mediated inhibition of NF-κB activation.

## Conclusions

In conclusion, the most important findings of this study demonstrate for the first time that the antiplatelet activity of sesamol may involve an increase in cAMP/PKA, followed by inhibition of NF-κB-PLC-PKC signaling events, which leads to inhibition of [Ca^2+^]i mobilization, and finally inhibition of platelet aggregation. Therefore, sesamol may represent an increased therapeutic potential to treat such thromboembolic disorders. Because platelet activation is not only linked to hemostasis, but also has a relevant role in inflammation and metastasis, our present data demonstrating that inhibition of NF-κB interferes with platelet function may have a great impact when these types of drugs are considered for treating cancer and various inflammatory diseases.

## Competing interests

The authors declare that they have no competing interests.

## Authors' contributions

CCC performed research and wrote the manuscript; WJL, ETO, CWC, and SCL performed the partial experiments and analyzed data; SYH and JRS conceived of the study and designed research. All authors read and approved the final manuscript.
